# Occurrence of *Bordetella* Infection in Pigs in Northern India

**DOI:** 10.1155/2014/238575

**Published:** 2014-01-12

**Authors:** Sandeep Kumar, Bhoj R. Singh, Monika Bhardwaj, Vidya Singh

**Affiliations:** Section of Epidemiology, Centre for Animal Disease Research and Diagnosis (CADRAD), Indian Veterinary Research Institute (IVRI), Izatnagar, Bareilly 243122, India

## Abstract

*Bordetella bronchiseptica* infection causing atrophic rhinitis in pigs is reported from almost all countries. In the present study, occurrence of *Bordetella* infection in apparently healthy pigs was determined in 392 pigs sampled to collect 358 serum samples and 316 nasal swabs from Northern India by conventional bacterioscopy, detection of antigen with multiplex polymerase chain reaction (mPCR), and detection of antibodies with microagglutination test (MAT) and enzyme linked immune-sorbent assay (ELISA). *Bordetella bronchiseptica* could be isolated from six (1.92%) nasal swabs. Although isolates varied significantly in their antimicrobial sensitivity, they had similar plasmid profile. The genus specific and species specific amplicons were detected from 8.2% and 4.4% nasal swabs using mPCR with *alc* gene (genus specific) and *fla* gene and *fim*2 gene (species specific) primers, respectively. Observations revealed that there may be other bordetellae infecting pigs because about 50% of the samples positive using mPCR for genus specific amplicons failed to confirm presence of *B. bronchiseptica*. Of the pig sera tested with MAT and ELISA for *Bordetella* antibodies, 67.6% and 86.3% samples, respectively, were positive. For antigen detection mPCR was more sensitive than conventional bacterioscopy while for detection of antibodies neither of the two tests (MAT and ELISA) had specificity in relation to antigen detection. Study indicated high prevalence of infection in swine herds in Northern India.

## 1. Introduction 

Bordetellosis caused by* Bordetella bronchiseptica *in pigs is an economically important disease, because infected pigs show a 6–10% reduced daily weight gain [1]. Although usually considered an opportunist or secondary invader [2], *B. bronchiseptica* can cause pneumonia and atrophic rhinitis in growing pigs [3–9]. Clinical signs of atrophic rhinitis or *B. bronchiseptica* infection are sneezing abnormal nasal discharge or epistaxis and shortening/deformity of snout. The pathogen is enzootic in many pig herds and pigs carry infection without any apparent signs of disease [10]. Bordetellosis in pigs is reported frequently from most of the countries; however, information is scant on *B. bronchiseptica* infection in pigs in India [11–13]. The disease is rarely reported in India except two outbreaks (on one farm) in Meghalaya, a North Eastern Indian State, in the recent past [12, 13]. Though piggery is much more common in other states of India, the disease is not reported. Besides a report of isolation from slaughtered pigs from Uttar Pradesh, North India [11], there is scanty information on prevalence of *B. bronchiseptica* pigs either as infection or a commensal. Therefore, the present study was planned with an aim of studying the occurrence of *Bordetella* infection in pigs in some parts of Northern India. The major question asked was does the pathogen exist in different states of India? To answer the question a few states with high density of pig population were selected for sampling to detect the status of *Bordetella* infection or the commensalism.

## 2. Material and Methods

### 2.1. Pig Samples

From a total of 392 pigs, 358 serum samples and 316 nasal swabs were collected from backyard and organized piggeries in Uttar Pradesh, Nagaland, and Haryana (Tables 1 and 2). All the serum samples were brought to the laboratory on ice within 12 to 36 h and all the nasal swabs were transferred to Amies Transport Media with charcoal (Hi-Media, Mumbai) and brought to laboratory at ambient temperature as soon as possible.

### 2.2. Conventional Bacteriology Methods for Isolation of *B. bronchiseptica* from Nasal Swabs

Swab samples were aseptically transferred to the five mL buffered peptone water (Difco, Saprks, USA) and incubated for 2–6 h at 37°C. Growth was streaked on blood agar (BA) and MacConkey lactose agar (MLA) plates. The suspected colonies (small, whitish, and slightly sticky) on BA were tested for catalase, oxidase production, and string formation with 10% KOH. Positive colonies were re-restreaked on brain heart infusion (BHI) agar (Difco) and incubated at 37°C for 24 hours. Pure cultures were tested for citrate utilization, nitrate reduction, reaction on triple sugar iron (TSI), hydrolysis of tween, motility, urease and indole production, lysine decarboxylation, and sugar fermentation. The isolates which utilized citrate as sole source of carbon, reduced nitrate to nitrite, produced urease but not fermented any sugar including glucose, lactose, sucrose, or salicin were suspected for *B. bronchiseptica* and further confirmed morphologically by Gram's staining and with mPCR. Reference strain of *B. bronchiseptica* (MTCC-6838) procured from IMTECH, Chandigarh, was used throughout the study as control.

### 2.3. Antimicrobial Susceptibility of *Bordetella bronchiseptica* Isolates

All *B*. *bronchiseptica* isolates were tested for antimicrobial sensitivity with disc diffusion method on Muller-Hinton (MHA) agar (Himedia) plates (CLSI, 2012) against amoxicillin (30 mcg), amoxicillin + clavulanic acid (30 mcg), azithromycin (30 mcg), aztreonam (30 mcg), cefotaxime (10 mcg), cefoxitin (30 mcg), ceftazidime (30 mcg), ceftazidime + clavulanic acid (30 + 10 mcg), ceftriaxone (30 mcg), ceftriaxone + sulbactam (30 + 15 mcg), ceftriaxone + tazobactam (30 + 10 *μ*g), chloramphenicol (10 mcg), ciprofloxacin (30 mcg), clindamycin (10 mcg), colistin (25 mcg), cotrimoxazole (25 mcg), doxycycline HCl (10 mcg), ertapenem (10 mcg), erythromycin (10 mcg), gentamicin (10 mcg), imipenem (10 mcg), lincomycin (10 mcg), nalidixic acid (30 mcg), nitrofurantoin (300 mcg), penicillin G, piperacillin + tazobactam (100 + 10 *μ*g), polymyxin B (50 U), roxithromycin (30 mcg), tetracycline (30 mcg), and vancomycin (30 mcg).

### 2.4. Plasmid Isolation

The standard culture, field isolate, and a reference multiplasmid *Escherichia coli* (E-382) strains were inoculated in 3 mL tryptic soya broth (TSB) incubated overnight at 37°C. Aliquot of 2 mL culture was used for plasmid isolation using QIAprep Spin Miniprep Kit (QIAGEN, Germany) as per instructions of the manufacturer. The plasmid DNA eluent was electrophoresed on agarose gel similar to PCR product described elsewhere.

### 2.5. Multiplex Polymerase Chain Reaction (mPCR)

#### 2.5.1. Template

For snap-chilled template, one mL aliquot from nasal swabs inoculated in buffered peptone water (for 2–6 h) was transferred to thin walled microcentrifuge tube and incubated at −20°C for fifteen min and then the tubes were transiently transferred to boiling water bath. Thereafter microcentrifuge tubes were centrifuged at 10000 rpm for ten min and supernatant was collected aseptically into fresh sterile vials and stored at −20°C till used for mPCR. Similarly, templets were also prepared from reference culture (MTCC-6838) and *B. bronchiseptica* isolates in the study.

#### 2.5.2. Primers

mPCR was standardized by using one genus (in house designed) and two species specific (Table 3) primers [14, 15]. The primers were designed by using bioinformatic tool. They were tested with standard strain before sample analysis. Primers were custom synthesized (Eurofins Genomic India Pvt. Ltd., Bangalore) and diluted to 10 pM/*μ*L in nuclease free water for use.

#### 2.5.3. Master Mix

For performing mPCR master mix was used. To make 900 *μ*L of master mix ingredients (Promega) were 100 *μ*L of 10 × PCR buffer, 100 *μ*L MgCl_2_ (25 mM), 60 *μ*L dNTPs (10 mM), 20 *μ*L each of the six primers (10 pM), 20 units of Taq polymerase, and nuclease free water to make the volume 900 *μ*L.

To perform mPCR, five *μ*L of template from snap-chilled supernatant was mixed with 45 *μ*L of master mix in a 200 *μ*L PCR tube. The PCR amplification was carried out with an initial denaturation at 95°C for ten min, denaturation of 35 cycles at 94°C for 30 s, annealing at 53°C for 30 s, extension at 72°C for 45 s, and a final extension at 72°C for seven min.

Ten *μ*L of mPCR products was mixed with 2 *μ*L of 6x ready to use gel loading dye (MBI, Fermentas) and run along with 100 bp DNA ladder (MBI, Fermentas) in 1% agarose gel (IBI Scientific, Peosta Lowa) containing 10 mg/mL ethidium bromide at 100 volts using 1x TBE electrophoresis buffer (Bio Basic Inc.). The gels were visualized and photographed under UV-gel documentation system (Alpha Innotech Co., USA).

Before multiplexing both the primer sets were used to perform uniplex PCR, but it was not done (to save the time and reagents) for all the samples because during standardization there was no difference between sensitivity and specificity of uniplex and multiplex PCR results.

The products of genus specific PCR, using in-house designed primers from two of the samples positive for genus specific PCR but not for species specific PCR, were sent for custom sequencing (Eurofins Genomic India Pvt. Ltd., Bangalore).

### 2.6. Determination of *Bordetella bronchiseptica* Antibodies in Pig Serum

#### 2.6.1. Preparation of Antigen for Microagglutination Test (MAT)

The method of Boot et al. [16] was followed to prepare phase I antigen from *B. bronchiseptica *(MTCC-6838). The stock antigen was diluted 1 : 20 in 0.5% formal saline to OD_269_ 0.5. The protein concentration was measured using Lowry's method for protein estimation (GeNei Protein Estimation Kit, Bangalore, India). The antigen was tested for autoagglutination and self-flocculation [17, 18]; flawless antigen was used in the study.

#### 2.6.2. MAT Procedure

The microagglutination test on dog sera was performed in U-bottom 96-well microtitre plates (Axygen Scientific, USA), using 150 *μ*L serum, twofold serially diluted in 150 *μ*L PBS, pH 7.2 (final dilution 1 : 2). The first and last row in each plate were used as positive and negative control, respectively. To each well, 150 *μ*L of diluted *B. bronchiseptica* antigen was added. Microtitre plates were incubated in a humid chamber to prevent drying of the plates and incubated for 48 h at 37°C. Plates were read against a dark background after 48 h [17]. The end point titre of MAT is defined as the final dilution of serum at which 50% of bordetellae are agglutinated. The reciprocal of the highest dilution up to which no button at bottom of the well in microplate was visible was considered as positive titre. Titre above and equal to 1 : 64 was considered as positive.

#### 2.6.3. Preparation of Antigen for ELISA

Antigen was prepared using the method of Boot et al. [17]. One standard and five field strains of *B. bronchiseptica* isolated from diverse sources were grown on BHI agar medium to harvest phase I antigen in PBS (with 0.1% merthiolate) and incubated overnight at room temperature. On the second day, the harvested culture was centrifuged at 6000 rpm for fifteen min. The supernatant was discarded and pellet was resuspended in merthiolated PBS and stored at 4°C as stock antigen. GeNei Protein Estimation Kit (Bangalore, India) was used to estimate protein concentration in whole cell antigen. The stock antigen was diluted in 0.1 M carbonate bicarbonate buffer (0.1 M, pH 9.6) to give final concentration of 10 *μ*g/mL.

#### 2.6.4. Protocol for ELISA

Antigen coated (each well with 100 *μ*L of 10 *μ*g/mL) were blocked for two h with 2% bovine serum albumin (BSA). Single dilution ELISA (1 : 200 diluted serums in PBS having 1% bovine serum albumin) was performed in triplicate. Goat-anti-pig-IgG alkaline phosphatase (Santacruz) conjugate was diluted 1 : 1500 in PBS with 1% bovine serum albumin and 100 *μ*L was used in each well. para-Nitrophenylphosphate, PNPP (Chem Cruz), dissolved (1 mg/mL) in carbonate buffer (0.1 M, pH 9.8) was used as substrate and plates were incubated in dark for 30 min after adding substrate. Then in each well 30 ul of 1 M NaOH was added to stop the reaction and plates were read with ELISA reader at 405 nm wavelength. For washing the plates at different steps PBS with 0.05% tween-20 was used.

*ELISA Titres Were Determined Using the: Following Formula*. ELISA titre = [(Average of test OD − average of negative control OD) ∗ 200]/Average of negative control OD.

ELISA titre values equal to or more than the half of the positive control (reference positive serum available in laboratory with titre of 300) were considered as positive for all inferences. A known negative serum giving no agglutination with the antigen and repeatedly yielding OD reading <0.070 was used as negative control.

Data was analysed using MS Excel worksheet using *χ* ^2^ test.

## 3. Result and Discussion

### 3.1. Bacteriological Results

On bacteriological analysis of 316 pig nasal swabs *B. bronchiseptica* could be isolated from six (1.92%) samples. There was no apparent effect of different media on isolation rate of *B. bronchiseptica*. Although growth was much better on blood agar than on MLA, *Proteus* often interfered with isolation on BA, and thus use of both media appeared to be satisfactory rather than one. The isolation rate in the present study varied from 0% to 3.6% from place to place. The lower isolation rate was reported earlier by many workers and may be due to geographic variation [18], type of sample used [13, 18, 19], overgrowth of the contaminants/commensal organisms on highly nutritious (BA) media [20, 21], and the different primary isolation media employed to isolate the pathogen [22]. All isolates of *B. bronchiseptica* were similar in morphological, growth, and biochemical characteristics. The pathogen was detected in nasal swabs of 2.2% grower and 2% adult pigs. No isolation of *B. bronchiseptica* from piglets might be due to passively acquired immunity from sows [23]. Consistent with this observation, in previous studies *B. bronchiseptica* infections have been reported in pigs mainly after the age of three weeks, when maternal immunity has waned [24]. In our study, out of six samples positive for *B. bronchiseptica*, 4 were from male (2.2%) and two were from females without any statistical significance (*P* > 0.1). Isolation rate of *B. bronchiseptica* from nasal swabs of backyard pigs was 1.3% and that of farmed pigs was 2.5% with no statistically significant difference (*P* > 0.1) between the two groups. From the single case of atrophic rhinitis observed during study both *Pasteurella multocida* type D and *B. bronchiseptica* were isolated indicating that mixed infection is required for precipitation of clinical disease [2, 6, 8]. No attempts were made to isolate *P. multocida* in the study to thoroughly concentrate on *B. bronchiseptica* so that occurrence of the pathogen in pigs in India may be determined.

### 3.2. Antimicrobial Sensitivity Assay

All *B. bronchiseptica* strains were resistant to amoxycillin, aztreonam, cefotaxime, ceftazidime, clindamycin, ceftazidime + clavulanic acid, cefoxitin, lincomycin, polymyxin B, penicillin, and vancomycin. Additionally, the strain isolated from pig of Nagaland was resistant for amoxicillin + clavulanic acid, while the isolate from a sample from Aligarh was resistant to ciprofloxacin, cotrimoxazole, nalidixic acid, and doxycycline. For other antimicrobials including azithromycin, colistin, ertapenem, imipenem, gentamicin, and piperacillin/tazobactam all the *B. bronchiseptica* strains were sensitive. Due to variation in antibiogram of isolates from different places no uniform antibiotic regimen may be recommended for treatment of bordetellosis in pigs. In china, Zhao et al. [25] reported similar variation (~40 antibiogram types) in antibiotic sensitivity pattern among *B. bronchiseptica* isolates from healthy pigs of different provinces.

### 3.3. Detection of *Bordetella* with mPCR

**Figure 1:**
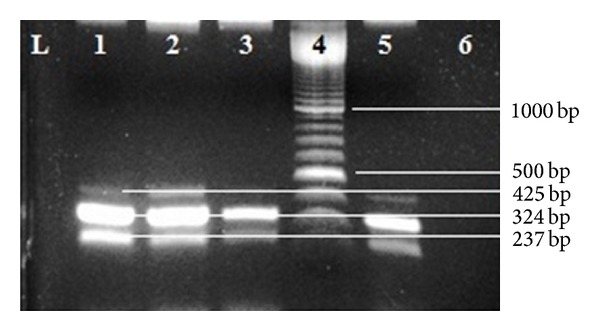
Multiplex PCR (mPCR) targeting *fim2 *(425 bp), *alc *(324 bp), and *fla *(237 bp) gene. Lane 1: positive control (*B. bronchiseptica* snap-chilled supernatant DNA); Lanes 2, 3, and 5: field sample (snap chilled supernatant); Lane 4: 100 bp ladder; Lane 6: negative control.

The mPCR standardized in the study had similar sensitivity and specificity as the uniplex PCR for genus and specific PCR. The mPCR standardized in the study was able to detect up to five cfu of *B. bronchiseptica* and three amplicons could be visualized in positive samples (Figure 1). A total of 14 samples were positive for all three amplicons while 12 for only genus specific amplicons. Customized sequencing of the PCR product from the two samples that were positive only for genus specific PCR showed the amplicons were 99% similar to the sequence of the *alc* gene of *Bordetella*, indicating the validity of the PCR results. Among the fourteen positive samples for *B. bronchiseptica* specific PCR, eight (3.9%) were from pigs on backyard piggeries and six (3.8%) were from pigs on organized piggeries with no significant (*P* > 0.1) difference among pigs reared under the two systems of piggery. None of the samples from indigenous pig was positive while samples from one large black Yorkshire and thirteen crossbred pigs were positive for *B. bronchiseptica* specific amplicon in PCR. Among the positive samples 7 each were from grower and adult animals but none from piglets. In previous studies too *B. bronchiseptica* infections have been reported mainly in pigs after the age of three weeks, when maternal immunity has waned [24]. Of the positives with PCR, nine samples were from male and 5 from female pigs showing significant difference in carriage of the pathogen by the animals of the two sexes (*P* < 0.10). Detection of *B. bronchiseptica *by PCR in a greater number of pigs than detected by culture indicated that PCR method was more sensitive than the conventional method, as reported earlier in several studies [14, 15]. Similar results have also been reported earlier in studies on *B. bronchiseptica* associated kennel cough in India [26]. Therefore, multiplex PCR may be recommended for rapid diagnosis of the *B. bronchiseptica* infection at an early stage and in carrier pigs.

### 3.4. *Bordetella* Antibody Detection

Only 6.7% of serum samples from Gurgaon (Haryana), 66.3% of samples from Nagaland, and 71.8% of samples from Uttar Pradesh were positive for *Bordetella bronchiseptica *agglutinins in MAT. Besides geography of the place of pig rearing, farming system appeared to be another important determinant for MAT positivity. Significantly (*P* < 0.10) more samples from backyard (84.5%) pigs were positive than the pigs reared on organized farms (44.7%). Earlier Attila-Lehel and Răpuntean [27] reported 42% positivity among growing and mature pigs keeping positive cutoff titre at 20. The higher rate of positivity in the present study might be due to geographic variation. Of the positive samples 115 (58.4%) were from male animals and 127 (78.9%) were from females (*P* < 0.00). A total of 34.2% piglets, 55% growers, and 90% adults were positive with MAT showing positive correlation of prevalence of *B. bronchiseptica *agglutinins with advancing age. Similarly, five (83.3%) indigenous, three (8.8%) large black Yorkshire, and 234 (73.6%) crossbred pigs sampled were positive with MAT for *Bordetella* agglutinins.

At cutoff titre (≥125), to have 100% sensitivity, 309 (86.3%) sera samples were positive for anti* B. bronchiseptica* IgGs with ELISA. Statewise, nine (60%) of Haryana, 70 (73.7%) of Nagaland, and 230 (92.7%) of Uttar Pradesh pig serum samples were positive. Among the positives, 182 (88.3%) samples were from pigs reared under backyard farming and 127 (83.6%) were from pigs under organized farming system with no significant effect of farming system (*P* > 0.10). Of 309 pig serum samples positive with ELISA, 170 (86.3%) were from male and 139 (86.3%) were from female pigs. Age appeared to be a significant determinant (*P* < 0.10) for positivity in ELISA. Positivity in ELISA was correlated with increasing age as only 63.2% piglets were positive in contrast to 88.2% and 92.1% positives among grower and adult pigs, respectively. Similar to age, breed was also a significant (*P* < 0.10) determinant for positivity in wc-ELISA as all the serum samples of indigenous pigs, 67.6% of large black Yorkshire, and 88.9% of crossbred pigs were positive with ELISA.

## 4. Conclusions

Isolation of the organism, though difficult, is the most authentic indication of infection and association with the disease. PCR is more sensitive than conventional bacterioscopy. Detection of the genus specific amplicon in 26 samples in contrast to species specific amplicon in fourteen indicated that *B. bronchiseptica* may not be the only *Bordetella* infecting pigs. ELISA has limited value to be used as diagnostic test, but MAT can be used for screening pig herd. More work is needed to determine diversity among bordetellae infecting pigs so that proper control measures can be undertaken.

## Figures and Tables

**Table 1:** Details of samples collected from pigs under different rearing systems.

Rearing system	Type of sample	Type of animals						Total (M/F)
		Piglet		Grower		Adult		
		M	F	M	F	M	F	
Backyard (215)	Only N	0	0	8	1	0	0	9 (8, 1)
	Only S	2	1	2	6	15	35	61 (19, 42)
	Both N and S	5	7	25	12	53	43	145 (83, 62)

Total		7	8	35	19	68	78	215 (110, 105)

Organized (177)	Only N	3	4	4	14	0	0	25 (7, 18)
	Only S	0	0	15	0	0	0	15 (15, 0)
	Both N and S	15	8	65	44	0	5	137 (80, 57)

Total		18	12	84	58	0	5	177 (102, 75)

Grand total (392)		25	20	119	77	68	83	392 (212, 180)

N: nasal swab; S: serum sample; P: piglet; G: grower; A: adult; M: male; F: female.

**Table 2:** Details of samples collected from pigs at different places.

State	Place	Pig breeds					
		Nondescript		Pure bred		Crossbred	
		M	F	M	F	M	F
Uttar Pradesh	Aligarh (56)	0	0	0	0	37	19
	Barabanki (12)	0	0	0	0	6	6
	IVRI, Bareilly (32)	0	0	0	0	20	12
	Abattoir, Bareilly (106)	2	3	0	0	66	35
	Chandpur, Bijnaur (16)	1	0	0	0	8	7
	Gajraula, Amroha (18)	0	0	0	0	5	13
	Meerut (34)	0	0	0	0	9	25

Nagaland	Akuluto (12)	0	0	0	0	5	7
	Jalukie (8)	0	0	0	0	4	4
	Jharnapani (28)	0	0	15	13	0	0
	Kohima (4)	0	0	0	0	2	2
	Merangkong (6)	0	0	0	0	2	4
	Saltazu (6)	0	0	0	0	2	4
	Tizit (8)	0	0	0	0	1	7
	Tuensang (12)	0	0	0	0	2	10
	Wokha (5)	0	0	0	0	1	4
	Dimapur (14)	0	0	9	5	0	0

Haryana	Gurgaon (15)	0	0	0	0	15	0

Total		3	3	24	18	185	159

M: male; F: female.

**Table 3:** Genus specific and species specific primers used in multiplex PCR for detection of *Bordetella bronchiseptica. *

Name of primers	Sequence 5′-3′	Product length (bp)	References
B688Bbalc-F	ACCAACCGCATTTATTCCTACTA	324	This study
B1012Bbalc-R	GGCCCTGGAGTTCGTATTTATG		

425BBfim-1 F	TGAACAATGGCGTGAAAGC	425	Xin et al., 2008 [15]
425BBfim-2 R	TCGATAGTAGGACGGGAGGAT		
237BBFla 4 F	TGGCGCCTGCCCTATC	237	Hozbor et al., 1999 [14]
237BBFla 2 R	AGGCTCCCAAGAGAGAAA		

F: forward primer; R: reverse primer.
